# Mesoporous Silica-Layered Gold Nanorod Core@Silver Shell Nanostructures for Intracellular SERS Imaging and Phototherapy

**DOI:** 10.3390/pharmaceutics16010137

**Published:** 2024-01-19

**Authors:** Sun-Hwa Seo, Ara Joe, Hyo-Won Han, Panchanathan Manivasagan, Eue-Soon Jang

**Affiliations:** Department of Applied Chemistry, Kumoh National Institute of Technology, Gumi 730-701, Gyeongbuk, Republic of Korea; 20126051@kumoh.ac.kr (S.-H.S.); jar@kumoh.ac.kr (A.J.); 20101414hyowon@kumoh.ac.kr (H.-W.H.); manimaribtech@gmail.com (P.M.)

**Keywords:** gold nanorod core, silver shell, cancer, phototherapy, SERS imaging

## Abstract

Precision diagnosis-guided efficient treatment is crucial to extending the lives of cancer patients. The integration of surface-enhanced Raman scattering (SERS) imaging and phototherapy into a single nanoplatform has been considered a more accurate diagnosis and treatment strategy for cancer nanotheranostics. Herein, we constructed a new type of mesoporous silica-layered gold nanorod core@silver shell nanostructures loaded with methylene blue (GNR@Ag@mSiO_2_-MB) as a multifunctional nanotheranostic agent for intracellular SERS imaging and phototherapy. The synthesized GNR@Ag@mSiO_2_-MB nanostructures possessed a uniform core–shell structure, strong near-infrared (NIR) absorbance, photothermal conversion efficiency (65%), dye loading ability, SERS signal, and Raman stability under phototherapy conditions. Under single 785 nm NIR laser irradiation, the intracellular GNR@Ag@mSiO_2_-MB nanostructures were dramatically decreased to <9%, which showed excellent photothermal and photodynamic effects toward cancer cell killing, indicating that the combination of photothermal therapy (PTT) and photodynamic therapy (PDT) of the GNR@Ag@mSiO_2_-MB nanostructures could greatly enhance the therapeutic efficacy of cancer cell death. GNR@Ag@mSiO_2_-MB nanostructures demonstrated a strong Raman signal at 450 and 502 cm^−1^, corresponding to the δ(C–N–C) mode, suggesting that the Raman bands of GNR@Ag@mSiO_2_-MB nanostructures were more efficient to detect CT-26 cell SERS imaging with high specificity. Our results indicate that GNR@Ag@mSiO_2_-MB nanostructures offer an excellent multifunctional nanotheranostic platform for SERS imaging and synergistic anticancer phototherapy in the future.

## 1. Introduction

Cancer is one of the most deadly diseases worldwide that kills millions of people every year [1]. Despite many efforts to develop new cancer nanotheranostics, the integration of diagnostic and therapeutic functions in an all-in-one single nanotheranostic platform is highly desired, which has recently emerged as a promising strategy for early diagnosis and therapy of cancer [2,3,4]. As a recently developed detection technique, SERS using plasmonic nanomaterials has emerged as a new and noninvasive imaging technique that has great promise to be applied as a powerful tool for cell detection and imaging of biological samples because of their high specificity, sensitivity, and low cost [5,6,7]. As a newly arising therapeutic strategy, phototherapy, such as PTT and PDT, has attracted widespread attention in recent years for the treatment of cancer because of its minimal invasion and high selectivity [8,9,10]. In PTT, the photothermal conversion materials convert NIR laser light into heat to destroy cancer cells [11,12]. In PDT, photosensitizers (PSs) absorb visible light or NIR light and convert molecular oxygen in the surrounding areas, generating cytotoxic reactive oxygen species (ROS), including singlet oxygen (^1^O_2_) to destroy cancer cells [13,14]. Thus, the combination of PTT/PDT functions with single NIR laser irradiation and cell SERS imaging into a single nanotheranostic platform is highly attractive and promising to achieve diagnostic, maximized therapeutic outcomes, and minimized recurrences. 

Plasmonic nanoparticles (NPs), especially gold nanorods (GNRs), have received considerable attention from biomedical researchers in the past few decades because of their unique properties, such as excellent biocompatibility, good stability, tunable NIR localized surface plasmon resonance (LSPR), photothermal heat conversion efficiency, and facile surface functionalization [15,16,17,18,19,20,21]. GNRs have also been employed as SERS active substrates for cancer cell imaging [22,23]. Silver (Ag) is another widely used plasmonic metal that demonstrates attractive size- and shape-dependent plasmonic properties and tunable LSPR [24]. The gold nanorod core@silver shell (GNR@Ag) is an excellent candidate for biomedical applications such as phototherapy and SERS imaging due to its high absorption from the ultraviolet (UV) to the NIR region by controlling its size or the Ag shell thickness [25]. However, the limited dye-loading capability of GNR@Ag with nonporous architectures severely restricts its usefulness in PDT [17,26]. Mesoporous silica (mSiO_2_) has been widely used as a coating material for GNR@Ag to enhance its dye-loading capability because of its large surface area, tunable size, and high pore volume [27,28]. Methylene blue (MB) is a photosensitizer (PS) that has been employed for PDT [29,30]. MB is well known to generate ROS, including ^1^O_2_ to destroy cancer cells under light irradiation [31]. MB was loaded into GNR@Ag@mSiO_2_ to form GNR@Ag@mSiO_2_-MB nanostructures [29]. Therefore, the combination of GNR@Ag@mSiO_2_-MB-based SERS imaging and phototherapy functions into a single nanoplatform is an innovative theranostic strategy for cancer therapy. To date, Wen et al. have developed a novel lysosome-targeted gold nanorod-cysteine-hydroxyl merocyanine for in situ SERS imaging and PTT [32]. Another study by Narayanan [8] reported that cucurbit uril can be regarded as glue for connection gold nanorods for SERS imaging and phototherapy [33]. To the best of our knowledge, there is no report about using GNR@Ag@mSiO_2_-MB nanostructures for intracellular SERS imagining and phototherapy. In this study, a new method was utilized to generate highly monodisperse and uniform GNR@Ag@mSiO_2_-MB nanostructures with strong NIR absorbance, photothermal conversion efficiency, large surface area, tunable size, dye loading ability, SERS signal, and Raman stability under phototherapy conditions, which were synthesized to develop a multifunctional nanosystem for cancer cell SERS imaging and phototherapy.

## 2. Materials and Methods

### 2.1. Chemicals and Reagents

All chemicals were obtained from Sigma-Aldrich (St. Louis, MO, USA) and were used as received without further purification.

### 2.2. Synthesis of Gold Nanorods

The gold nanorod (GNR) solution was prepared using a silver (Ag)-catalyzed and seed-mediated method in a binary surfactant system [34]. In brief, a gold seed solution was prepared by mixing 5 mL of 0.2 M hexadecyltrimethylammonium bromide (CTAB; ≥99%) aqueous solution with 5 mL of 0.5 mM gold(III) chloride trihydrate (HAuCl_4_⋅3H_2_O; ≥99.9%) before adding 0.6 mL of cold 10 mM sodium borohydride (NaBH_4_; ≥99.99%) to the solution and vortexing the mixture for 3 min. The resultant brownish-yellow seed solution was aged at 25 °C for 30 min. The growth solution was prepared by adding 100 mL of 1 mM HAuCl_4_⋅3H_2_O to 50 mL of 0.15 M CTAB solution and 50 mL of 0.1 M benzyldimethylhexadecylammonium chloride (BDAC; ≥99%) solution. Subsequently, a solution containing 5 mL of 4 mM silver nitrate (≥99.0%) and 1.4 mL of 7.9 mM L-ascorbic acid (AA; ≥99.0%) was added to the mixture under magnetic stirring, with the result that the yellow solution became colorless. Finally, 0.24 mL of the seed solution was gradually added while stirring the growth solution, resulting in dark-red color changes within a few hours. After the resulting solution was left to further react at 25 °C for 12 h, the excess reagents were removed by centrifugation and washed with deionized water (DI).

### 2.3. Synthesis of GNR Core@Ag Shell Nanostructure

A silver (Ag) shell was created on the surface of the GNR core using a procedure developed by the Niidome group [35]. In brief, a 0.08 M cetyltrimethylammonium chloride (CTAC) solution was mixed with 10 mL of the GNR aqueous solution. The pH of the resulting mixture was adjusted to 5.4 by combining it with 0.4 mL of 0.5 M NaOH and 2 mL of 0.1 M AA solution, and subsequently, 0.1 mL of 0.01 M AgCl solution was slowly added under magnetic stirring. The resulting solution (GNR@Ag) was further incubated at 25 °C for 3 h, which led to a color change from red to green. The excess reagents were removed from the final solution by centrifugation and washed with DI.

### 2.4. Synthesis of GNR Core@mSiO_2_ Shell Nanostructure

mSiO_2_ layer was coated on the as-prepared GNRs using a cationic surfactant-mediated method [36]. Briefly, 10 mL of the GNR solution was added with anhydrous ethanol (4 mL) and DI (6 mL). After aging for 3 h, 200 μL of 0.1 M NaOH solution and 60 μL of 20% tetraethylorthosilicate (TEOS) in EtOH were gently stirred and injected three times for 30 min intervals. The resultant solutions were then kept at 25 °C for two days while undergoing vigorous stirring. Finally, the product (GNR@mSiO_2_) was purified by centrifugation and washed with DI water.

### 2.5. Preparation of GNR Core@Ag@mSiO_2_ Shell Nanostructure

GNR@Ag@mSiO_2_ samples were synthesized using two different procedures. The first approach involved coating the surface of the GNR@Ag with mSiO_2_ (procedure (1)). The procedure was identical to that of the GNR@mSiO_2_ synthesis, with the only difference being the use of a GNR@Ag solution instead of a GNR solution. The second method involved the diffusion of Ag^+^ ions into the mSiO_2_ layer of GNR@mSiO_2_ using a concentration gradient, and this was followed by the formation of an Ag layer on the surface of the GNRs (procedure (2)). Procedure (2) was the same as that used to prepare GNR@Ag, except for the use of a GNR@mSiO_2_ solution instead of a PEGylated GNR solution.

### 2.6. Synthesis of Methylene Blue (MB)-Loaded GNR@Ag@mSiO_2_

The GNR@Ag@mSiO_2_ aqueous solution (10 mL) was combined with an excess of MB dye (1 mg) under magnetic stirring at 25 °C for 24 h. During the reaction, to prevent the photodegradation of the MB dye, the mixture was protected from exposure to external light using aluminum foil. The resultant products underwent several centrifugations and DI water washes until the supernatant became colorless, which was resuspended in DI water and then lyophilized (GNR@Ag@mSiO_2_-MB).

### 2.7. Characterization

The morphology of all nanostructures was characterized via transmission electron microscopy (TEM; JEOL, JEM2100, Tokyo, Japan). The morphology and elemental composition of GNR@Ag@mSiO_2_-MB were analyzed using field-emission TEM (FETEM) and scanning TEM (STEM) in combination with energy dispersive X-ray spectroscopy (EDX) and mapping (JEM-ARM200F, JEOL, Tokyo, Japan). Optical absorption was performed on an Optizen 3220 UV–visible spectrophotometer (Mecasys, Daejeon, Republic of Korea). Micro-Raman analysis using 785 nm excitation (laser power = 2 μW; spot area = 51 μm^2^; accumulation = 5 times) was obtained using an inVia Raman microscope system (Renishaw, Wotton-under-Edge, UK) equipped with a Leica DM 2500 microscope (Leica, Wetzlar, Germany) using a 1200 g/mm grating. We investigated the LSPR properties of the plasmon hybridization-based GNR@Ag using a numerical simulation method. The gold (Au) content was accomplished using inductively coupled plasma optical emission spectroscopy (ICP-OES, USA).

### 2.8. Measurement of Photothermal Properties

To measure the photothermal effect of nanomaterials, a single 785 nm NIR laser (power = 0.6 W/cm^2^; 600 s) was used to irradiate the GNR, GNR@mSiO_2_, GNR@Ag@mSiO_2_, and GNR@Ag@mSiO_2_-MB aqueous solutions with concentrations of 100 μg/mL. During NIR laser irradiation, the temperature monitoring of the dispersed NP solutions was captured using an infrared (IR)–thermal camera (SE/A325, FLIR Systems Inc., Wilsonville, OR, USA) controlled by a PC application. Furthermore, the gold (Au) concentration of the GNR@Ag@mSiO_2_-MB was measured to 100 µg/mL, and the GNR@Ag@mSiO_2_-MB suspension at different volumes (12, 16, 20, and 24 µL of 100 µg/mL) was exposed to a single 785 nm NIR laser (0.6 W/cm^2^) for 600 s. The temperature variation was captured by an IR-thermal camera. The photothermal stability of GNR@Ag@mSiO_2_-MB (24 µL of 100 µg/mL) solution was recorded by continuous five cycles of NIR laser irradiation and natural cooling. The photothermal conversion efficiency (*η*) of GNR@Ag@mSiO_2_-MB (24 µL of 100 µg/mL) can be calculated according to Equation (1) [37,38].

(1) $$\eta =\frac{hS\left(T_{Max}-T_{Sur}\right)-Q_{dis}}{I(1-10^{-A_{785}})}$$

### 2.9. Cell Culture and In Vitro Phototherapy

The CT-26 murine colorectal carcinoma cell line was obtained from the Korean Cell Line Bank (KCLB). The CT-26 cells were cultured in Dulbecco’s modified Eagle’s medium (DMEM) containing 10% fetal bovine serum (FBS) and 100 units/mL of penicillin–streptomycin at 37 °C with 5% CO_2_. Subsequently, the CT-26 cells were seeded in a 48-well culture dish at a density of 6 × 10^4^ cells/well and incubated overnight. After injecting the different concentrations of GNR, GNR@mSiO_2_, GNR@Ag@mSiO_2_, MB, and GNR@Ag@mSiO_2_-MB (0–24 µL of 100 µg/mL) in culture media into each well, the CT-26 cells were further cultured for 8 h and were or were not exposed to a single 785 nm NIR laser (0.6 W/cm^2^) for 600 s. After incubating for 6 h, the cellular viability was assessed via 3-(4,5)-dimethylthiahiazo-2-yl-2,5-diphenyltetrazolium bromide (MTT) assay. Furthermore, CT-26 cells were cultured in a 48-well culture dish for 24 h. The cells were then treated with GNR@Ag@mSiO_2_, MB, and GNR@Ag@mSiO_2_-MB at the same concentrations (24 µL of 100 µg/mL) for 8 h. Subsequently, the cells were exposed to 785 nm laser (0.6 W/cm^2^) for 600 s or 660 nm laser (0.6 W/cm^2^) for 600 s or the combination of 785/660 nm two lasers irradiation (0.6 W/cm^2^) for 600 s. After laser irradiation, the cells were incubated for another 6 h, and the cell viability was then assessed by MTT assay.

To assess and visualize the intracellular phototherapy activity of GNR, GNR@mSiO_2_, GNR@Ag@mSiO_2_, and GNR@Ag@mSiO_2_-MB, CT-26 cells were co-stained with calcein-acetoxymethyl ester (calcein-AM) and propidium iodide (PI) solution of a live/dead double staining kit using a Nikon A1R confocal laser scanning microscope (Nikon, Tokyo, Japan). CT-26 cells were cultured in 35 mm covered glass-bottom culture plates for 24 h. CT-26 cells were incubated with GNR, GNR@mSiO_2_, GNR@Ag@mSiO_2_, and GNR@Ag@mSiO_2_-MB (24 µL of 100 µg/mL) for 8 h, were or were not exposed to 785 nm laser (0.6 W/cm^2^) for 600 s and then incubated for 6 h. Finally, CT-26 cells were stained and imaged using a confocal laser scanning microscope.

2′,7′-Dichlorodihydrofluorescein diacetate (H_2_DCFDA) can serve as a fluorescent probe for the detection of photodynamic therapy (PDT)-stimulated intracellular ROS generation that enters the cells and interacts with reactive oxygen to generate strong green fluorescence in the presence of ROS. Based on this, CT-26 cells were cultured in 35 mm covered glass-bottom culture plates overnight. Then, CT-26 cells were incubated with GNR, GNR@mSiO_2_, GNR@Ag@mSiO_2_, and GNR@Ag@mSiO_2_-MB (24 µL of 100 µg/mL) for 8 h and were or were not exposed to 785 nm laser (0.6 W/cm^2^) for 600 s. After incubating for another 6 h, CT-26 cells were stained with H_2_DCFDA (40 µM) for 30 min, which were captured on a confocal laser scanning microscope.

### 2.10. Cell SERS Imaging

To explore the temperature changes and ROS between cells and the temperature distribution and ROS generation inside a single cell during cell death induced by phototherapy, cell SERS imaging was performed using an inVia Raman microscope system (Renishaw, Wotton-under-Edge, UK) equipped with a Leica DM 2500 microscope (Leica, Wetzlar, Germany) using a 1200 g/mm grating and a 785 nm NIR laser (power = 2 μW; spot area = 51 μm^2^; accumulation = 5 times) beam directed to the sample via a 20× objective lens. For cell SERS mapping experiments, CT-26 cells were cultured into glass slides for 24 h. CT-26 cells were treated with MB, GNR@mSiO_2_-MB, and GNR@Ag@mSiO_2_-MB for 8 h and subsequently were exposed to 785 nm NIR laser (power = 2 μW; spot area = 51 μm^2^; accumulation = 5 times) for 600 s. The laser spot was adjusted to completely cover the area of each slide. The slides were fixed with 4% paraformaldehyde solution and rinsed with DI water. After that, the glass slides were performed for cell SERS mapping using an inVia Raman microscope system.

### 2.11. Statistical Analysis

Data are represented as mean ± standard deviation (SD). Statistical analysis was measured using a one-way analysis of variance by SPSS 23.0 software. * *p* < 0.05 and ** *p* < 0.01 were regarded as statistically significant.

## 3. Results and Discussion

### 3.1. Synthesis and Characterization of GNR@Ag@mSiO_2_-MB

**Scheme 1:**
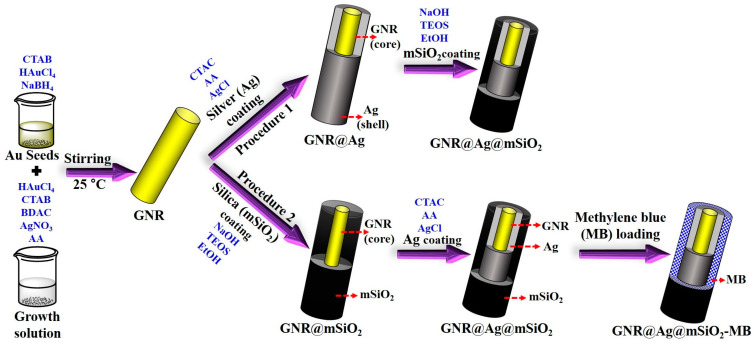
Schematic illustration of two different procedures for the synthesis of GNR@Ag@mSiO_2_ and GNR@Ag@mSiO_2_-MB nanostructures.

**Figure 1:**
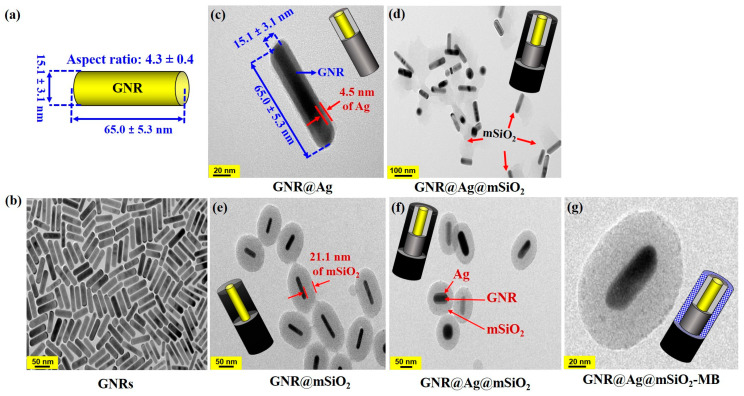
TEM images of two different procedures for the synthesis of GNRs (**a**,**b**), GNR@Ag (**c**), GNR@mSiO_2_ (**e**), GNR@Ag@mSiO_2_ (**d**,**f**), and GNR@Ag@mSiO_2_-MB nanostructures (**g**).

The schematic diagram of two different procedures for the synthesis of GNR@Ag@mSiO_2_ and GNR@Ag@mSiO_2_-MB nanostructures is shown in Scheme 1. The first procedure involved coating the surface of the GNR@Ag with mSiO_2_ to form GNR@Ag@mSiO_2_ nanostructures. The second procedure involved the diffusion of Ag^+^ ions into the mSiO_2_ layer of GNR@mSiO_2_ using a concentration gradient, and this was followed by the formation of an Ag layer on the surface of the GNRs and then MB was loaded into GNR@Ag@mSiO_2_ to form GNR@Ag@mSiO_2_-MB nanostructures. Figure 1 shows TEM images of the prepared GNR@Ag@mSiO_2_ and GNR@Ag@mSiO_2_-MB nanostructures obtained using the two different procedures. As shown in Figure 1a,b, the GNRs as a raw material exhibit excellent well-defined rod structures with an average length and width of 65.0 ± 5.3 nm and 15.1 ± 3.1 nm, respectively, yielding an aspect ratio of 4.3 ± 0.4. In procedure (1), GNR@Ag@mSiO_2_ was synthesized from GNR@Ag using a mSiO_2_ coating process. As shown in Figure 1c, a 4.5 nm thick Ag layer was homogeneously coated onto the GNRs. However, the mSiO_2_ layer did not uniformly coat the GNR@Ag surface and seemed to be preferentially coated on one side, as indicated by the arrows in Figure 1d. In our previous study, we successfully created a uniform mSiO_2_ layer on GNRs by adjusting the ratio of EtOH to H_2_O within the range of 0.25 to 0.5 [39]. At this optimized ratio of EtOH to H_2_O, an EtOH-rich phase developed in the hydrophobic tail region of the CTAB bilayer attached to the GNR surface because of the variance in the dielectric constants of EtOH (*ε* = 25.3) and H_2_O (*ε* = 80.1), whereas a water-rich phase was generated in the hydrophilic head group. Therefore, hydrophobic TEOS, as a silica precursor, tends to diffuse preferentially into the lipophilic hydrocarbon chain region with the EtOH-rich phase of the CTAB bilayer [39,40]. In other words, the presence of a homogeneous surfactant bilayer on the GNR surface is crucial for the formation of a uniform mSiO_2_ layer [41]. This indicates that in the current study, the partial elimination of the CTAC surfactant from certain GNR@Ag surfaces during the washing procedure might have led to the asymmetric formation of the mSiO_2_ layer.

**Figure 2:**
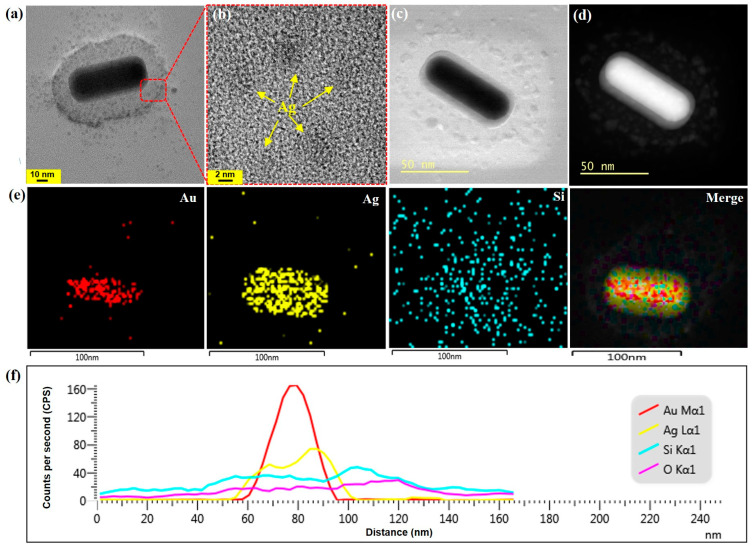
(**a**,**b**) FETEM images of GNR@Ag@mSiO_2_-MB. Bright-field (**c**) and dark-field (**d**) STEM images of GNR@Ag@mSiO_2_-MB. EDX elemental mapping (**e**) and spectroscopy (**f**) of GNR@Ag@mSiO_2_-MB.

Using procedure (2), GNR@Ag@mSiO_2_ was derived from GNR@mSiO_2_ via Ag layer-coating. Although the same silica coating procedure was used to coat GNR@Ag, when GNR was coated, a homogeneous mSiO_2_ layer was created on the GNR surface, as shown in Figure 1e. This implies that the CTAB bilayer remained intact on the GNR surface even after washing, serving as a stable template for the formation of the mSiO_2_ layer. Furthermore, the Ag layer was selectively coated only on the GNR core within the GNR@mSiO_2_ structure, as indicated by the arrow in Figure 1f. We believe that the Ag monolayer was created on the GNR surface through the underpotential deposition of Ag^+^ ions, which facilitated the preferential growth of the Ag layer. Finally, MB was loaded into mSiO^2^ with a high loading capability to form GNR@Ag@mSiO_2_-MB nanostructures (Figure 1g). Additionally, the morphologies of GNR@Ag@mSiO_2_-MB were further characterized by FETEM and STEM. The FETEM image of GNR@Ag@mSiO_2_-MB was uniformly coated with Ag and mSiO_2_ thicknesses of 4.5 nm and 21.1 nm, respectively (Figure 2a). As shown in the FETEM image in Figure 2b, it was confirmed that Ag quantum dots (QDs) of approximately 4.0 nm in size were crystallized not only in the mSiO_2_ layer but also around it. Furthermore, the bright-field (Figure 2c) and dark-field STEM images (Figure 2d) confirm that the bright Ag QDs are closer to the Ag layer than the mSiO_2_ layer. The formation of GNR@Ag@mSiO_2_-MB nanostructure was further verified via energy-dispersive X-ray (EDX) elemental mapping (Figure 2e) and spectroscopy (Figure 2f), confirming that Ag and Si were uniformly distributed at distances of approximately 5.2 and 20.5 nm, respectively, from the surface of the GNR. Nonetheless, the EDX intensity of the Ag detected between the Ag and mSiO_2_ layers was almost negligible, indicating that the amount of Ag QDs on the mSiO_2_ layer was significantly smaller than that in the Ag layer on the surface of the GNR.

**Figure 3:**
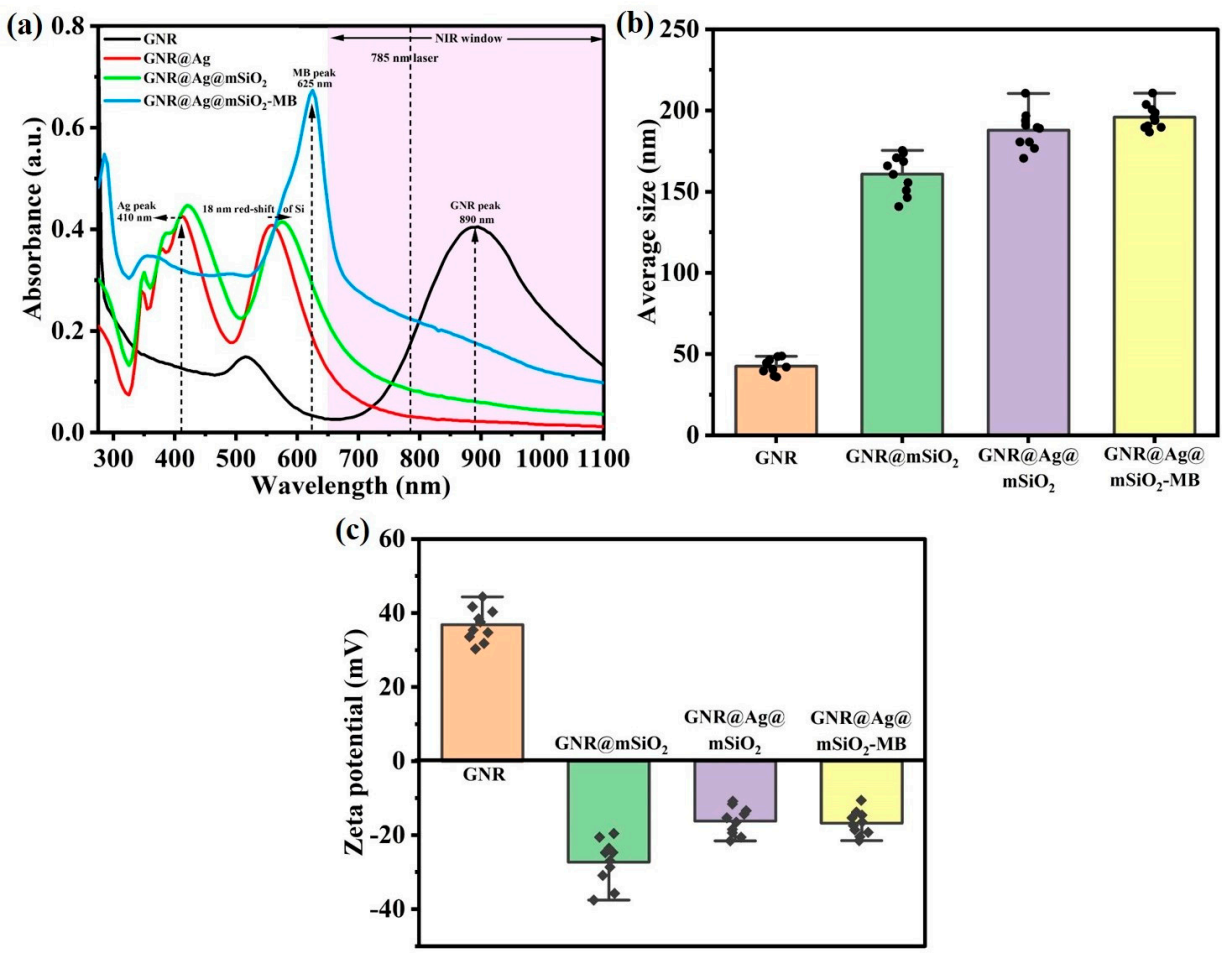
(**a**) UV–vis absorption spectra of GNR, GNR@Ag, GNR@Ag@mSiO_2_, and GNR@Ag@mSiO_2_ nanostructure. Size distribution histogram (**b**) and zeta potential (**c**) of GNR, GNR@Ag, GNR@Ag@mSiO_2_, and GNR@Ag@mSiO_2_ nanostructure.

The optical properties of GNR, GNR@Ag, GNR@Ag@mSiO_2_, and GNR@Ag@mSiO_2_ nanostructure were recorded using an UV–vis spectrophotometer (Figure 3a). GNR exhibits a strong longitudinal surface plasmon resonance (*λ*_LSPR_) band in the NIR region, which makes it suitable for PTT [42]. After layering with an Ag shell, the Ag peak was observed at 410 and the *λ*_LSPR_ peak of GNR@Ag had a slight blue shift to 557 nm, which is different from the original GNR. Subsequent surface coating with mSiO_2_ and GNR@Ag@mSiO_2_ gives rise to a small redshift at 575 nm. After MB loading, the UV–vis absorption spectra of GNR@Ag@mSiO_2_-MB exhibited an absorption peak of MB at 625 nm and a slight red shift in the NIR region, suggesting that MB was successfully loaded. The particle size and surface charge of GNR, GNR@mSiO_2_, GNR@Ag@mSiO_2_, and GNR@Ag@mSiO_2_-MB were recorded via DLS (Figure 3b) and zeta potential (ZP) (Figure 3c). The particle size distributions of GNR, GNR@mSiO_2_, GNR@Ag@mSiO_2_, and GNR@Ag@mSiO_2_-MB nanostructures were determined to be 41.5 ± 2.3 nm, 161.1 ± 4.7 nm, 187.0 ± 1.7 nm, and 195.4 ± 3.5 nm, respectively. Additionally, the ZP of GNR, GNR@mSiO_2_, GNR@Ag@mSiO_2_, and GNR@Ag@mSiO_2_-MB were +36.8 ± 0.8 mV, −27.5 ± 0.6 mV, −16.4 ± 1.0 mV, and −16.9 ± 0.4 mV, respectively, suggesting that GNR was coated with various functional materials.

### 3.2. Changes in LSPR Absorption of GNR@Ag@mSiO_2_ upon Increasing Ag Layer Thickness

**Figure 4:**
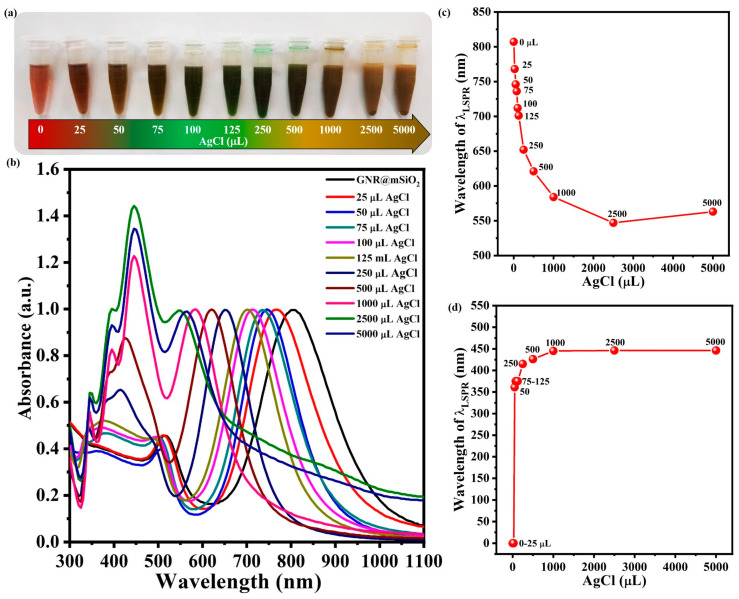
Photographic images (**a**), UV–vis absorption spectra (**b**), and variation in *λ*_LSPR_ for the GNR core (**c**) and the Ag shell (**d**) of GNR@Ag@mSiO_2_ samples obtained using different volumes of 0.01 M AgCl solution from 25 to 5000 μL.

Figure 4a shows photographic images of the GNR@Ag@mSiO_2_ prepared by adding 0 to 5000 μL of 0.01 M AgCl solution to the GNR@mSiO_2_ solution. The resultant solution changed from wine red to green and finally to dark brown as the concentration of AgCl increased, and the absorption wavelength of the *λ*_LSPR_ of the GNR blue shifted from 807 to 547 nm (Figure 4b,c). However, the *λ*_LSPR_ of the Ag layer in GNR@Ag@mSiO_2_ was red-shifted from 361 to 446 nm when the AgCl concentration was increased from 50 to 5000 μL (Figure 4b,d). This indicates that the GNR@Ag aspect ratio in the mSiO_2_ layer decreased as the Ag layer thickness increased.

**Figure 5:**
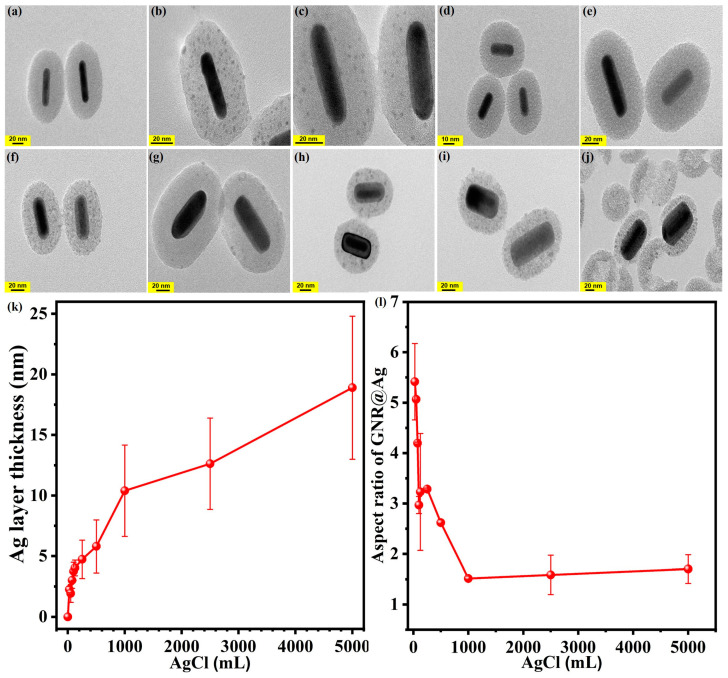
TEM images of GNR@Ag@mSiO_2_ synthesized using different volumes of 0.01 M AgCl aqueous solutions: (**a**) 25, (**b**) 50, (**c**) 75, (**d**) 100, (**e**) 125, (**f**) 250, (**g**) 500, (**h**) 1000, (**i**) 2500, and (**j**) 5000 μL. Variation in Ag layer thickness (**k**) and aspect ratio (**l**) of GNR@Ag within mSiO_2_ layer with the volume of AgCl solution used in the synthesis.

Figure 5a–j shows TEM images of GNR@Ag@mSiO_2_ prepared using different volumes of AgCl solution in the range of 2–5000 μL. Significant differences among the samples were observed not only in the lateral thickness of the Ag layer but also in the aspect ratio of GNR@Ag, as shown in Figure 5k,l. Specifically, within the AgCl solution volume range from 0 to 1000 μL, the aspect ratio of the GNR@Ag rapidly decreased from 4.0 ± 1.4 to 2.1 ± 1.0 as the lateral thickness of the Ag layer increased from 0 to 10.4 ± 3.8 nm. However, for AgCl solution volumes of greater than 1000 μL of AgCl, the change in the GNR@Ag aspect ratio was minimal as (2.0 ± 0.4), even though the lateral thickness of the Ag layer increased to 18.9 ± 5.9 nm. This indicates that the growth rate of the lateral Ag layer is faster than that in the longitudinal direction when the volume of AgCl in the growth solution is between 0 to 1000 μL. However, with the use of AgCl solution volumes greater than 1000 μL, the growth rates in the two directions become similar.

**Figure 6:**
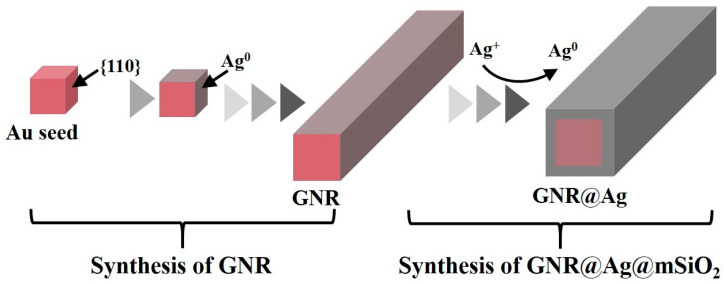
Anisotropic growth mechanism of the Ag shell on the GNR core.

As shown in Figure 6, Ag^+^ ions, acting as catalysts for GNR synthesis, protect the {110} facets of the GNRs. Consequently, it is possible that the {110} facets coated by Ag^+^ ions or Ag monolayers play a catalytic role in the further growth of the Ag layer on the GNR, resulting in faster lateral growth of the Ag layer in GNR@Ag@mSiO_2_. However, using more than 2500 μL of AgCl solution led to the partial destruction of the mSiO_2_ layer due to the excessive thickness of the Ag layer in GNR@Ag@mSiO_2_.

**Figure 7:**
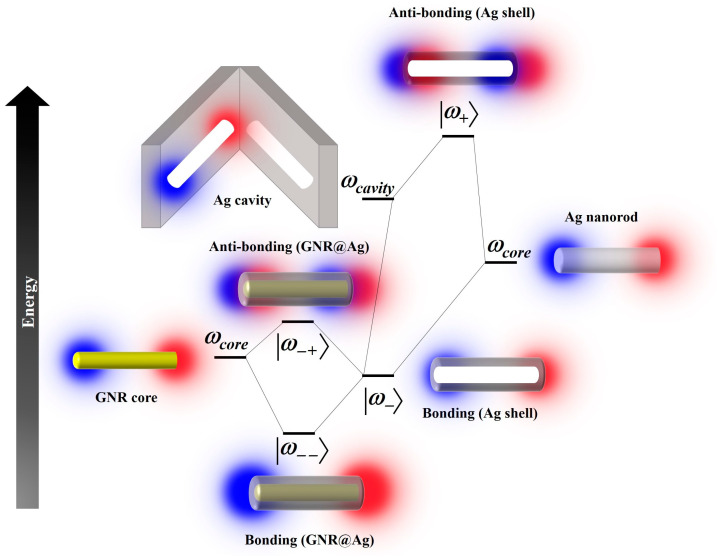
Energy-level diagram illustrating plasmon hybridization in GNR@Ag. Blue and red shaded circles represent negative and positive surface charges induced by electromagnetic excitation.

To further investigate the origin and shift of the absorption peak with the increase in the shell thickness, we analyze the surface plasmon modes using the plasmon hybridization mechanism to explain the absorption band shift. The plasmon response of the Ag shell can be used as an interaction between an Ag core (ω_core_) mode and an Ag cavity (ω_cavity_) mode [43]. As illustrated in Figure 7, a hollow Ag shell exhibits a symmetrically coupled plasmon mode (or bonding mode, |ω_−_〉) with a lower energy and an anti-symmetrically coupled plasmon mode (or antibonding mode, |ω_+_〉) with a higher energy owing to the hybridization of Ag cavity and Ag nanorod plasmons [44,45,46,47]. The strength of the coupling interaction between the Ag cavity and Ag nanorod plasmons is controlled by the thickness of the Ag shell [44]. For GNR@Ag in the mSiO_2_ layer, hybridization between the GNR core plasmon and the bonding plasmon of the Ag shell leads to the generation of bonding (|ω_− −_〉) and anti-bonding (|ω_− +_〉) modes. This plasmonic hybridization efficiently suppresses the interband damping of the Ag shell because the plasmon resonance energy of the GNR core is higher than that of the bonding mode of the Ag shell [44]. Therefore, there are three dipolar plasmon resonances for the GNR@Ag, such as antibonding mode (|ω_+_〉), bonding (|ω_− −_〉) mode, and antibonding (|ω_− +_〉) mode, respectively. Figure 7 exhibits the energy of these hybridized plasmon modes as a function of the Ag shell thickness, indicating that Ag shell thickness increased because of their blue shift of the lower energy mode (|ω_−_ 〉) and also observed blue shifts in the absorption surface plasmon resonance (SPR) peak because of their antibonding (|ω_− +_〉) mode (Figure 5b) [48].

### 3.3. SERS Effect of GNR@Ag@mSiO_2_-MB

**Figure 8:**
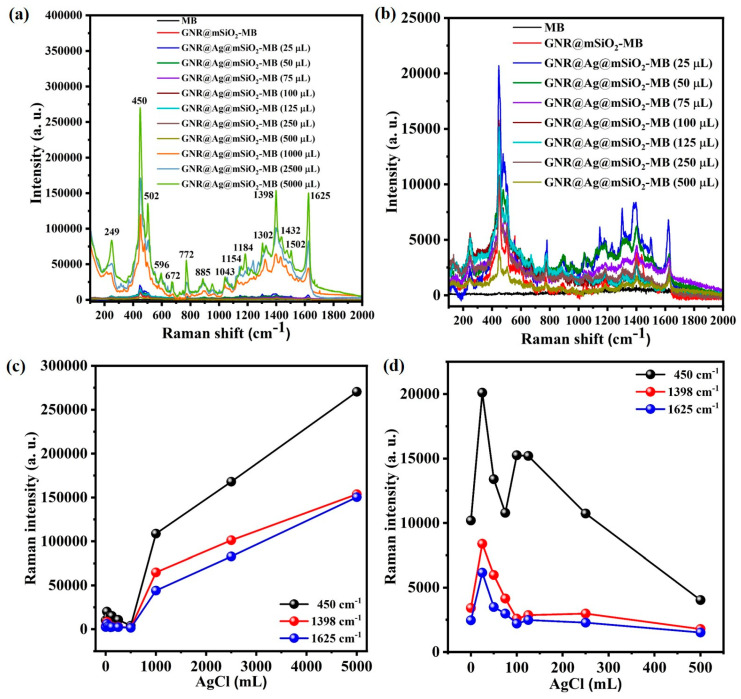
Micro-Raman spectra (laser wavelength = 785 nm; power = 2 μW; spot area = 51 μm^2^) of MB, GNR@mSiO_2_-MB, and GNR@Ag@mSiO_2_ synthesized using different volumes of 0.01 M AgCl solution: (**a**) 25–5000 μL and (**b**) 25–500 μL. Change in Raman intensity of δ(C–N–C), ν(C–N), and ν(C–C)_ring_ modes at 450, 1398, and 1625 cm^−1^ for GNR@Ag@mSiO_2_-MB synthesized different volumes of 0.01 M AgCl solution: (**c**) 25–5000 μL and (**d**) 25–500 μL.

Figure 8a,b compare the micro-Raman spectra of GNR@Ag@mSiO_2_-MB with different Ag layer thicknesses with that of the MB solid film and GNR@mSiO_2_-MB acquired using a 785 nm excitation laser (power = 2 μW; spot area = 51 μm^2^; ND filter = 10^−4^%). In a previous study, we measured the Raman spectra of MB-GNR@mSiO_2_ using an excitation laser power of 70 μW [49]. However, the Raman intensity of the GNR@Ag@mSiO_2_-MB sample synthesized using more than 1000 μL of the AgCl solution (hereinafter referred to as GNR@Ag@mSiO_2_-MB (1000 μL)) was remarkably strong, exceeding the detection limit. Consequently, we reduced the excitation laser power to 2 μW for the Raman analysis in the present study. Table 1 lists the characteristic MB Raman band assignments observed in the spectra of the GNR@Ag@mSiO_2_-MB nanostructure [50,51,52].

As shown in Figure 8b, there are no Raman bands in the spectrum of the solid MB film, which can be attributed to the difference between the excitation laser wavelength (785 nm) and absorption wavelength (655 nm) of MB. In contrast, the Raman intensities of the Au–N stretching mode (ν(Au–N)) at 249 cm^−1^, the C–N–C skeletal deformation mode (δ(C–N–C)) at 450 cm^−1^, the C–H out-of-plane bending mode (γ(C–H)) at 772 cm^−1^, the C–N symmetric stretching mode (ν(C–N)) at 1398 cm^−1^, and the C–C ring stretching mode (ν(C–C)_ring_) at 1625 cm^−1^ in the spectra of the GNR@Ag@mSiO_2_-MB nanostructure increased significantly with the thickness of the Ag layer. In particular, the observation of the ν(Au–N) mode indicates that the MB molecules loaded onto the mSiO_2_ layer were bonded to the GNR@Ag surface [49,50].

Figure 8c shows the variation in the intensities of the δ(C–N–C), ν(C–N), and ν(C–C)_ring_ modes for the GNR@Ag@mSiO_2_-MB samples prepared using different volumes of AgCl solution; these Raman band intensities increased significantly with the AgCl solution volume in the range of 1000 to 5000 μL. Interestingly, within the AgCl solution volume range of 0 to 500 μL, the intensity of the three aforementioned Raman modes of GNR@Ag@mSiO_2_-MB was maximized when 25 μL of AgCl solution was used (Figure 8d). However, these intensities decreased when the volume of AgCl was larger or smaller than 25 μL. According to Zhang et al., the coupling interaction between the Ag cavity and Ag nanorod plasmons is strongest when the thickness of the Ag shell is 2.0 nm [44]. This results in the plasmon energy of the bonding mode for the Ag shell being lower than that of the GNR core. Consequently, the splitting energy between the bonding and anti-bonding modes in the GNR@Ag nanostructure increases, leading to the amplification of the LSPR in this nanostructure. The Ag shell thickness of GNR@Ag@mSiO_2_-MB synthesized using 25 μL of AgCl solution appears to be approximately 2.0 nm (Figure 5a), which explains the increase in Raman intensity for the nanostructures prepared using 25 μL of AgCl solution. However, there is a significant increase in the Raman intensity once again when the Ag shell thickness exceeds 10.2 nm (corresponding to AgCl volumes of 1000 μL or more). This indicates that the splitting energy between the |ω_− −_〉 and |ω_− +_〉 modes for GNR@Ag increased remarkably owing to the enhanced strength of the coupling between the Ag cavity and Ag-nanorod plasmons when the Ag-layer thickness exceeded 10.2 nm. However, additional theoretical studies are required to explain these phenomena.

### 3.4. Measurement of Photothermal Properties

**Figure 9:**
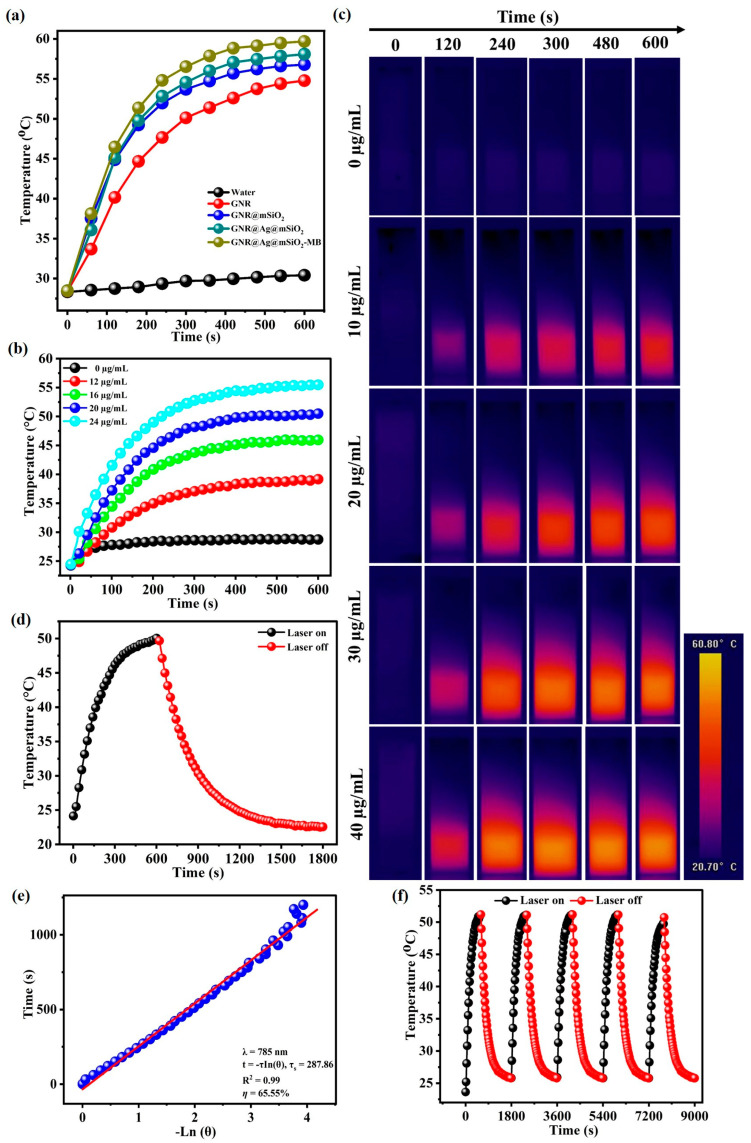
(**a**) The photothermal heating curves of GNR, GNR@mSiO_2_, GNR@Ag@mSiO_2_, and GNR@Ag@mSiO_2_-MB suspensions under 785 nm laser irradiation (0.6 W/cm^2^) for 600 s. The photothermal heating profiles (**b**) and IR thermal images (**c**) of GNR@Ag@mSiO_2_-MB suspension with different volumes (12, 16, 20, and 24 µL of 100 µg/mL) under 785 nm laser irradiation (0.6 W/cm^2^) for 600 s. (**d**) The photothermal heating curves of GNR@Ag@mSiO_2_-MB under 785 nm (0.6 W/cm^2^) for 600 s, followed by natural cooling down with the laser turning off for 1200 s. (**e**) Plots of the cooling period versus the negative natural logarithm of driving force temperature. (**f**) Repeated heating/cooling profiles for five cycles.

To explore the photothermal effect of NPs, GNR, GNR@mSiO_2_, GNR@Ag@mSiO_2_, and GNR@Ag@mSiO_2_-MB aqueous solutions with concentrations of 100 μg/mL were irradiated by a single 785 nm NIR laser (0.6 W/cm^2^) for 600 s (Figure 9a). The photothermal heating curves of GNR, GNR@mSiO_2_, GNR@Ag@mSiO_2_, and GNR@Ag@mSiO_2_-MB exhibited a strong concentration and laser power density-dependent photothermal efficacy with the maximum temperature increment up to 54.78 °C, 56.78 °C, 58.09 °C, and 59.68 °C. In contrast, the temperature of pure water increased by only 30.41 °C. In addition, the Au concentration of the GNR@Ag@mSiO_2_-MB was measured to be 100 µg/mL, and the GNR@Ag@mSiO_2_-MB suspension with different volumes (12, 16, 20, and 24 µL of 100 µg/mL) was exposed to a 785 nm laser (0.6 W/cm^2^) for 600 s (Figure 9b). The photothermal heating curves of GNR@Ag@mSiO_2_-MB (24 µL/mL) solutions increased to 55.47 °C, which is high enough to kill cancer cells. By contrast, the photothermal heating curves of pure water increased by only 28.74 °C, which is not enough to kill cancer cells. The photothermal heating curves were captured using an IR–thermal camera (Figure 9c). To investigate the photothermal conversion efficiency of GNR@Ag@mSiO_2_-MB, the solution was irradiated by a single 785 nm NIR laser (0.6 W/cm^2^) for 600 s, and the temperature was obtained followed by natural cooling down with laser turning off for 1200 s (Figure 9d). The photothermal conversion efficiency (*η*) of GNR@Ag@mSiO_2_-MB (24 µL of 100 µg/mL) was found to be 65%, indicating that the GNR@Ag@mSiO_2_-MB showed the potential to act as an effective photothermal material (Figure 9e). Furthermore, the photothermal stability of GNR@Ag@mSiO_2_-MB (24 µL of 100 µg/mL) was assessed by repeating the lasering and cooling cycles five times. As seen in Figure 9f, no noticeable variations in the temperature profiles were recorded for GNR@Ag@mSiO_2_-MB after repeating five cycles, suggesting that GNR@Ag@mSiO_2_-MB possessed excellent photothermal stability.

### 3.5. In Vitro Phototherapy

In order to evaluate the in vitro phototherapy using a single 785 nm NIR-absorbing nanostructures, cytotoxicity was assessed using the MTT assay. Figure S1 displays the results of the MTT assay for CT-26 cells treated with GNR, GNR@mSiO_2_, GNR@Ag@mSiO_2_, MB, and GNR@Ag@mSiO_2_-MB at different concentrations (0–24 µL of 100 µg/mL) for 8 h and CT-26 cells were or were not exposed to a single 785 nm NIR laser (0.6 W/cm^2^) for 600 s. After incubating for another 6 h, the cell viability was above 70% at high concentrations of all nanostructures without a single 785 nm NIR laser irradiation, suggesting very low cytotoxic effects of all nanostructures. In contrast, cell viability decreased to less than 9% when GNR@Ag@mSiO_2_-MB (24 µL of 100 µg/mL) were treated with CT-26 cells and to a single 785 nm NIR laser (0.6 W/cm^2^) for 600 s, suggesting the effectiveness of GNR@Ag@mSiO_2_-MB as promising phototherapy agents [49].

To further investigate the in vitro phototherapy using double 785/660 nm laser irradiation, cell viability was evaluated by MTT assay (Figure S2). The cell viability of GNR@Ag@mSiO_2_-MB (24 µL of 100 µg/mL) was dramatically decreased to 5%, which was triggered by a double 785 nm NIR laser (0.6 W/cm^2^) for 600 s and 660 nm laser (0.6 W/cm^2^) for 600 s and showed the excellent synergistic PTT and PDT effects of GNR@Ag@mSiO_2_-MB nanostructures. However, combined 785/660 nm two lasers generally suffer from inevitable systemic side effects because of the use of two different irradiation wavelengths for PTT and PDT [53]. To solve this issue, it is highly desirable to develop a novel single NIR laser-induced multifunctional nanostructure for synergistic PTT/PDT. The cell viability of GNR@Ag@mSiO_2_-MB (24 µL of 100 µg/mL) nanostructures was also significantly decreased to <9% after a single 785 nm NIR laser irradiation for 600 s, which was slightly lower than double laser irradiation (785/660 nm laser). Similarly, Phan et al. (2017) fabricated polypyrrole-MB NPs for an effective combination of PTT and PDT using a single 808 nm NIR laser irradiation [54]. Another example, Fan et al. developed an MB-bound nanoplatform for synergistic PTT/PDT using a single 785 nm NIR laser irradiation [55].

**Figure 10:**
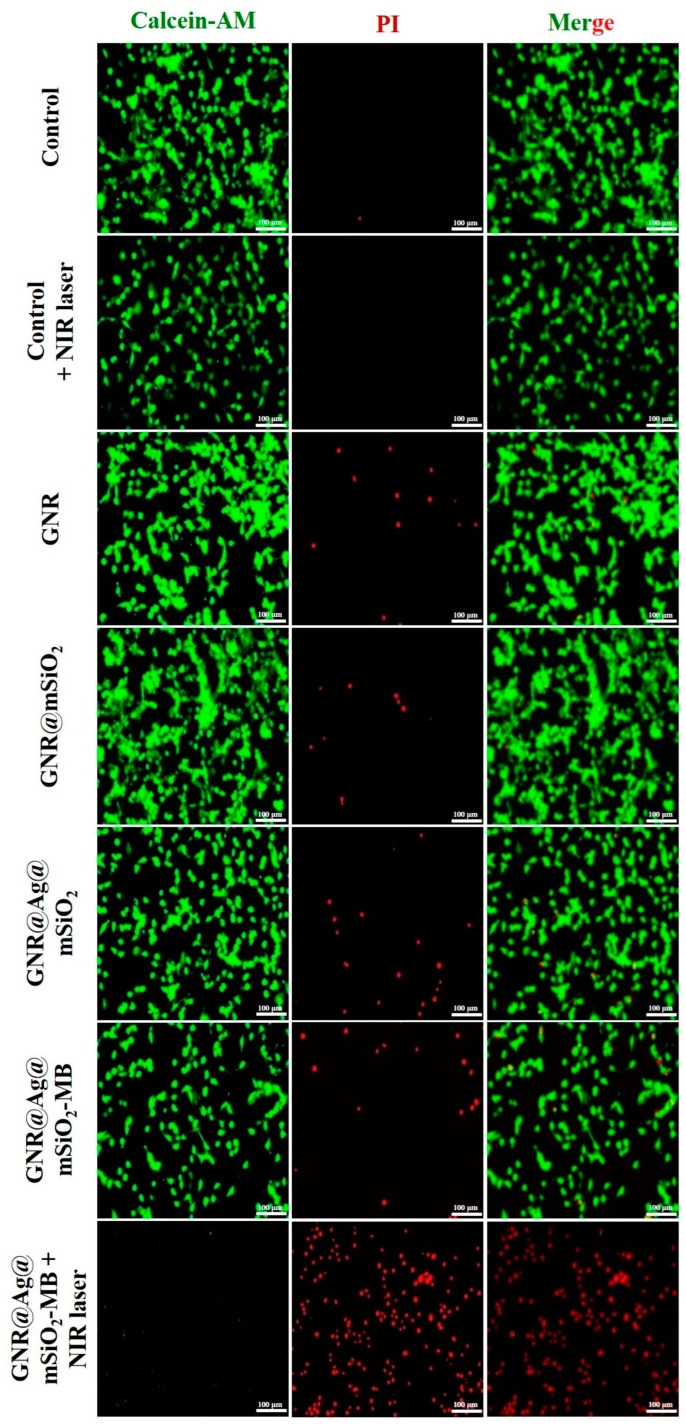
Confocal fluorescence images of CT-26 cells stained with calcein-AM/PI under various treatment groups such as control, control + NIR laser, GNR only, GNR@mSiO_2_ only, GNR@Ag@mSiO_2_ only, GNR@Ag@mSiO_2_-MB only, and GNR@Ag@mSiO_2_-MB + NIR laser (20× magnification; scale bar: 100 μm).

In order to direct observation of the intracellular photothermal activity of GNR, GNR@mSiO_2_, GNR@Ag@mSiO_2_, and GNR@Ag@mSiO_2_-MB (24 µL of 100 µg/mL), CT-26 cells were stained with calcein-AM (live, green fluorescence) and PI (dead, red fluorescence), respectively (Figure 10). The strong green fluorescences (viable cells) were found in control and control + NIR laser. GNR, GNR@mSiO_2_, GNR@Ag@mSiO_2_, and GNR@Ag@mSiO_2_-MB-treated groups without NIR laser irradiation showed negligible cell death. In contrast, strong red fluorescence (cell death) was observed in the GNR@Ag@mSiO_2_-MB + NIR laser irradiation, suggesting the excellent photothermal effect of the designed GNR@Ag@mSiO_2_-MB [49,50].

To further investigate the photodynamic activity of GNR, GNR@mSiO_2_, GNR@Ag@mSiO_2_, and GNR@Ag@mSiO_2_-MB (24 µL of 100 µg/mL), CT-26 cells were stained with 2′,7′-dichlorodihydrofluorescein diacetate (H_2_DCFDA). As shown in Figure S3, no fluorescence was found in control groups, and the negligible green fluorescence signal was found in the GNR, GNR@mSiO_2_, GNR@Ag@mSiO_2_, and GNR@Ag@mSiO_2_-MB-treated groups without NIR laser irradiation, By contrast, CT-26 cells treated with GNR@Ag@mSiO_2_-MB + NIR laser irradiation exhibit strong green fluorescence, suggesting an amount of ROS production in cells.

### 3.6. Cell SERS Imaging

**Figure 11:**
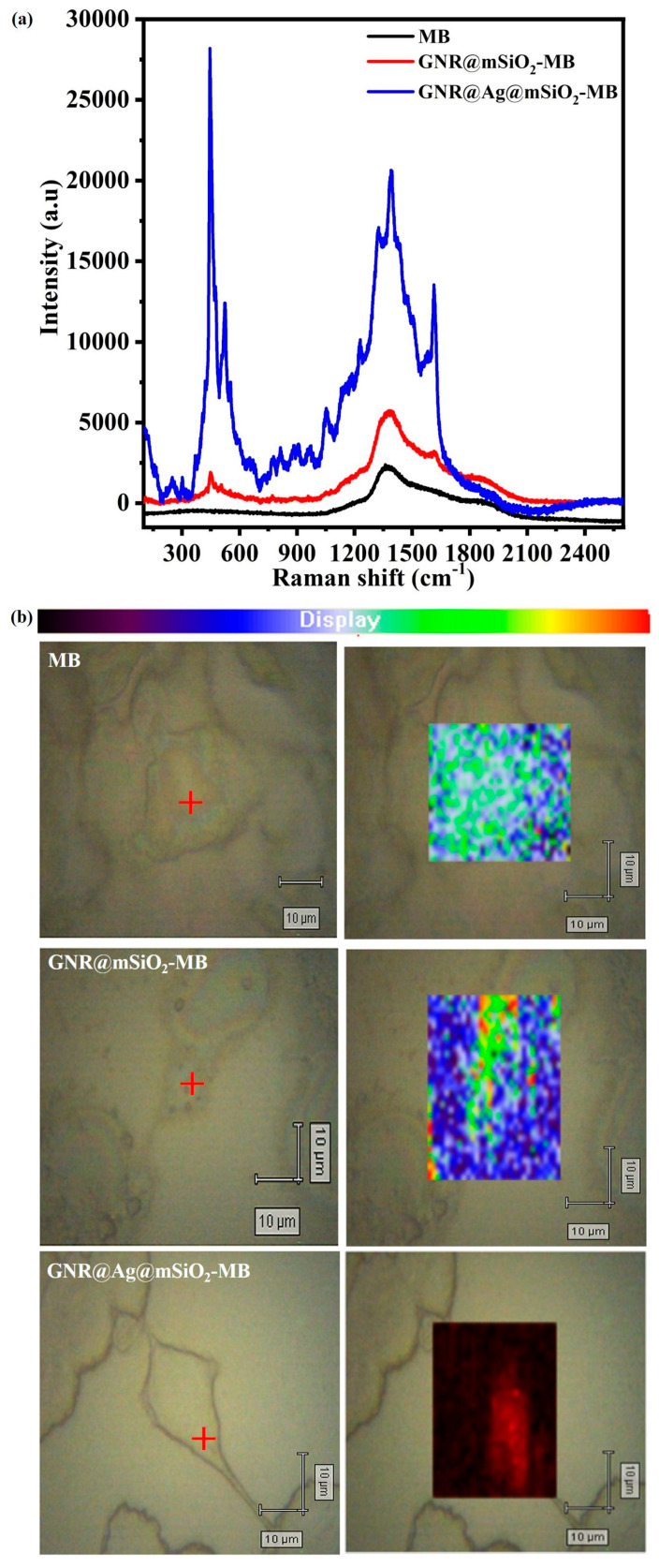
Micro-Raman spectra and merged bright-field/Raman mapping images of CT-26 cells treated with MB, GNR@mSiO_2_-MB, and GNR@Ag@mSiO_2_-MB. The color contrast in the mapping images corresponds to the intensity of the δ(C–N–C) vibrational band of MB. The red cross in the bright-field images shows the NIR laser excitation site corresponding to the spectra shown on the left (laser wavelength = 785 nm; power = 2 μW; spot area = 51 μm^2^).

In order to achieve the real-time monitoring of microscopic temperature changes during in vitro phototherapy, SERS-based monitoring techniques using nanomaterials in theranostics have recently received considerable attention in the biomedical field because of their ultrahigh sensitivity, multiplex ability, and photostability, which have great promise for accurate cancer detection [5,56]. For cell SERS mapping experiments, CT-26 cells were treated with MB, GNR@mSiO_2_-MB, and GNR@Ag@mSiO_2_-MB for 8 h and subsequently were exposed to 785 nm NIR laser (power = 2 μW; spot area = 51 μm^2^; accumulation = 5 times) for 600 s. Figure 11a,b shows micro-Raman spectra and the mapping images of CT-26 cells treated with MB, GNR@mSiO_2_-MB, and GNR@Ag@mSiO_2_-MB. The Raman intensity of the δ(C–N–C) mode of GNR@Ag@mSiO_2_-MB-treated CT-26 cells was 15 times stronger than GNR@mSiO_2_-MB-treated CT-26 cells, indicating that the Raman peaks of GNR@Ag@mSiO_2_-MB were more efficient to detect cancer. In contrast, no Raman peaks were observed in the MB-treated CT-26 cells. The cell SERS images demonstrate that GNR@Ag@mSiO_2_-MB-treated CT-26 cells exhibit good performance in SERS imaging, indicating that the SERS imaging can clearly reveal the distribution of NPs for image-guided therapy. The laser spots indicated by the red cross in Figure 11b exhibited obvious brightness differences in the SERs mapping, implying that SERS imaging-guided phototherapy is advantageous for a better understanding of biological processes occurring within a cell and precise treatment.

## 4. Conclusions

In summary, we developed a novel method to produce monodisperse and uniform GNR@Ag@mSiO_2_-MB nanostructures for intracellular SERS imaging and phototherapy. The as-prepared GNR@Ag@mSiO_2_-MB nanostructures possessed high stability, broad NIR absorbance, photothermal stability, dye loading ability, strong SERS effect, and low toxicity. More importantly, GNR@Ag@mSiO_2_-MB nanostructures exhibited outstanding photothermal conversion efficiency of approximately 65%, which was significantly higher than that of previously reported photothermal agents. The GNR@Ag@mSiO_2_-MB nanostructures showed excellent SERS activities under 785 NIR laser excitation, which have served as an efficient contrast agent for the rapid SERS imaging of cancer cells. The PTT/PDT of GNR@Ag@mSiO_2_-MB nanostructures could synergistically kill cancer cells via a low power density (0.6 W/cm^2^) of 785 nm NIR laser for 600 s, indicating that GNR@Ag@mSiO_2_-MB nanostructures have superior photothermal conversion efficiency and excellent ROS-generating ability. Therefore, we demonstrated that the GNR@Ag@mSiO_2_-MB nanostructures hold tremendous potential as a new theranostic system for SERS imaging and phototherapy. Despite its potential, SERS imaging-guided phototherapy is still in the laboratory stage. Up to now, there have been no reports of clinical trials involving SERS imaging-guided phototherapy. Hence, the emerging SERS imaging-guided phototherapy using NPs had several issues and difficulties, and advances in science and technology might also promote the clinical translation of SERS imaging-guided phototherapy.

## Figures and Tables

**Table 1:** Raman frequencies and corresponding assignments for characteristic bands observed in the spectra of the GNR@Ag@mSiO_2_-MB solution excited using a near-infrared laser (*λ* = 785 nm).

Assignment	Frequency (cm^−1^)
ν(Au–N)	249
δ(C–N–C)	450
δ(C–N–C)	502
δ(C–S–C)	596
γ(C–H)	672
	772
	885
	953
β(C–H)	1043
	1154
ν(C–N)	1184
ν(N–CH_3_)	1225
	1302
	1324
ν_sym_(C–N)	1398
ν_asym_(C–N)	1432
ν_asym_(C–C)_ring_	1471
	1502
ν(C–C)_ring_	1625

## Data Availability

The data presented in this study are available in this article and supplementary material.

## Supplementary Materials

The following supporting information can be downloaded at: https://www.mdpi.com/article/10.3390/pharmaceutics16010137/s1, Figure S1. MTT assay results for CT-26 cells incubated with GNR, GNR@mSiO_2_, GNR@Ag@mSiO_2,_ and GNR@Ag@mSiO_2_-MB at various concentrations (0-24 µL of 100 μg /mL) with or without NIR laser irradiation (0.6 W/cm^2^) for 600 s; Figure S2. MTT assay results for CT-26 cells incubated with GNR@Ag@mSiO_2,_ MB, and GNR@Ag@mSiO_2_-MB at the same concentrations (24 µL of 100 μg /mL) with or without 785 nm laser (0.6 W/cm^2^) for 600 s or 660 nm laser (0.6 W/cm^2^) for 600 s or the combination of 785/660 nm two lasers irradiation (0.6 W/cm^2^) for 600 s; Figure S3. Confocal fluorescence images of ROS production in CT-26 cells stained with H_2_DCFDA under various treatment groups such as control, control + NIR laser, GNR only, GNR@mSiO_2_ only, GNR@Ag@mSiO_2_ only, GNR@Ag@mSiO_2_-MB only, and GNR@Ag@mSiO_2_-MB + NIR laser (20× magnification; scale bar: 100 μm).
