# Supplementary material for: Mesoporous Silica-Layered Gold Nanorod Core@Silver Shell Nanostructures for Intracellular SERS Imaging and Phototherapy
Source: Pharmaceutics. 2024 Jan 19;16(1):137. doi: 10.3390/pharmaceutics16010137 (PMC10821141; doi:10.3390/pharmaceutics16010137)
Supplement: Supplementary file 1 [file pharmaceutics-16-00137-s001.zip › pharmaceutics-2797461-supplementary.pdf]

Mesoporous Silica-Layered Gold Nanorod Core@Silver Shell Nanostructures for Intracellular SERS Imaging and Phototherapy

Sun-Hwa Seo †, Ara Joe †, Hyo-Won Han, Panchanathan Manivasagan and Eue-Soon Jang *

Department of Applied Chemistry, Kumoh National Institute of Technology, Gumi 730-701, Gyeongbuk, Republic of Korea; 20126051@kumoh.ac.kr (S.-H.S.); manimaribtech@gmail.com (P.M.); jar@kumoh.ac.kr (A.J.); 20101414hyowon@kumoh.ac.kr (H.-W.H.)

* Correspondence: euesoon@kumoh.ac.kr

† These authors contributed equally to this work.

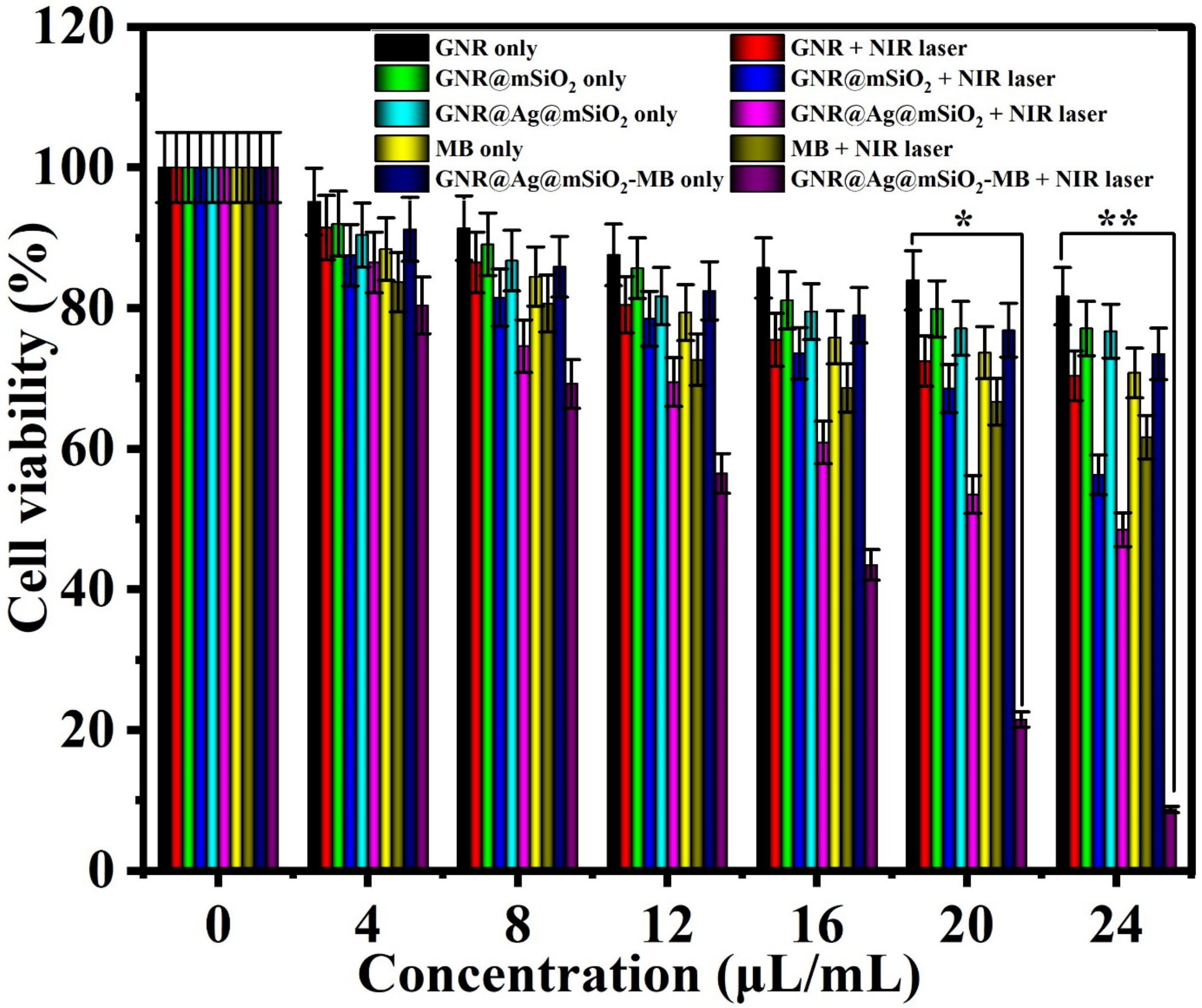

Figure S1. MTT assay results for CT-26 cells incubated with GNR, GNR@mSiO_2, GNR@Ag@mSiO_2, and GNR@Ag@mSiO_2-MB at various concentrations (0-24 μL of 100 μg /mL) with or without NIR laser irradiation (0.6 W/cm^2) for 600 s. Data were represented as mean ± standard deviation (SD). A * $p < 0.05$ and ** $p < 0.01$ were regarded as statistically significant.

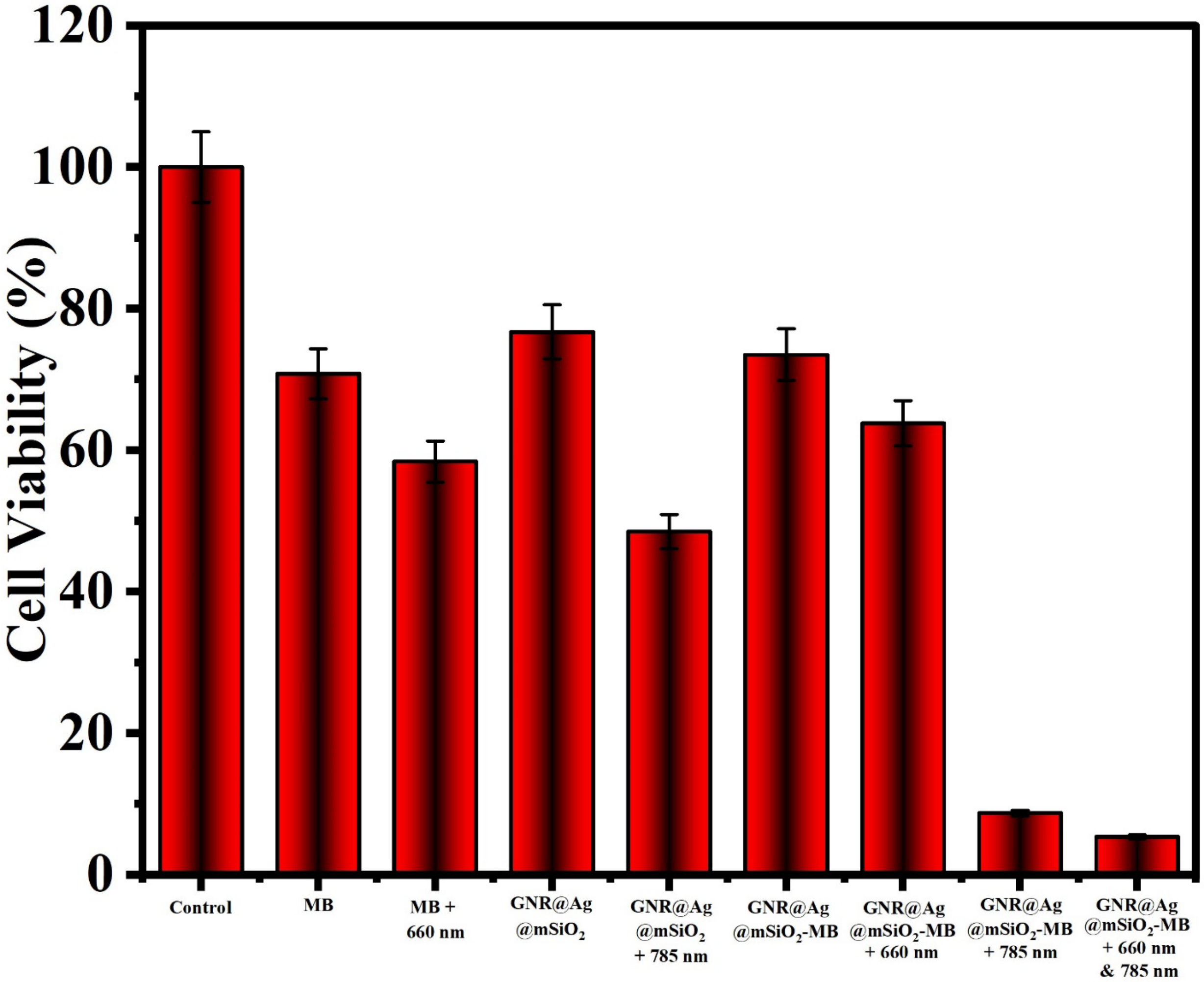

Figure. S2. MTT assay results for CT-26 cells incubated with GNR@Ag@mSiO$_2$, MB, and GNR@Ag@mSiO$_2$-MB at the same concentrations (24 μL of 100 μg /mL) with or without 785 nm laser (0.6 W/cm^2) for 600 s or 660 nm laser (0.6 W/cm^2) for 600 s or the combination of 785/660 nm two lasers irradiation (0.6 W/cm^2) for 600 s. Data were represented as mean ± standard deviation (SD).

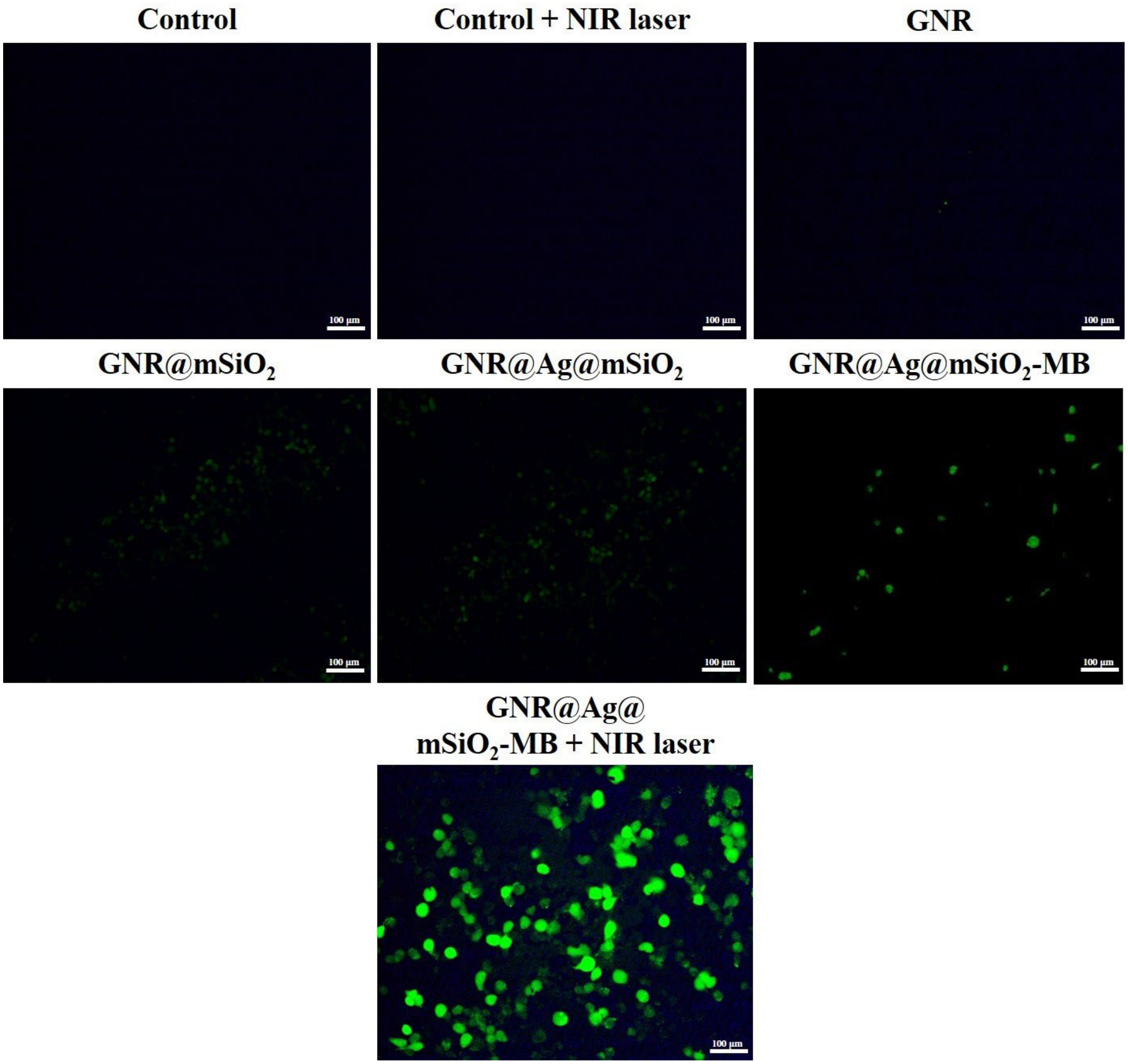

Figure S3. Confocal fluorescence images of ROS production in CT-26 cells stained with H_2DCFDA under various treatment groups such as control, control + NIR laser, GNR only, GNR@mSiO_2 only, GNR@Ag@mSiO_2 only, GNR@Ag@mSiO_2-MB only, and GNR@Ag@mSiO_2-MB + NIR laser (20 × magnification; scale bar: 100 μm).
